# Pregnancy complications in acquired thrombotic thrombocytopenic purpura: a case–control study

**DOI:** 10.1186/s13023-014-0193-6

**Published:** 2014-11-28

**Authors:** Barbara Ferrari, Alberto Maino, Luca A Lotta, Andrea Artoni, Silvia Pontiggia, Silvia M Trisolini, Alessandra Malato, Frits R Rosendaal, Flora Peyvandi

**Affiliations:** Fondazione IRCCS Ca’ Granda Ospedale Maggiore Policlinico Milano, Angelo Bianchi Bonomi Hemophilia and Thrombosis Centre, Milan, Italy; Cellular Biotechnologies and Hematology, Sapienza University, Rome, Italy; UOC di Ematologia con UTMO, Ospedali Riuniti Villa Sofia-Cervello, Palermo, Italy; Department of Clinical Epidemiology, Leiden University Medical Center, Leiden, The Netherlands; Department of Thrombosis and Haemostasis, Leiden University Medical Center, Leiden, The Netherlands; Department of Pathophysiology and Transplantation, Università degli Studi di Milano, Milan, Italy

**Keywords:** ADAMTS13, Anti-ADAMTS13 antibodies, Miscarriage, Pregnancy, Thrombotic thrombocytopenic purpura

## Abstract

**Background:**

Pregnant women with a history of acquired thrombotic thrombocytopenic purpura (TTP) are considered at risk for disease recurrence and might be at risk for miscarriage, similar to other autoimmune disorders. However, the exact entity of these risks and their causes are unknown. The aim of this study was to evaluate risk factors associated with adverse pregnancy outcome, in terms of both gravidic TTP and miscarriage, in women affected by previous acquired TTP.

**Methods:**

We conducted a nested case–control study in women with a history of acquired TTP enrolled in the Milan TTP registry from 1994 to October 2012, with strict inclusion criteria to reduce referral and selection bias.

**Results:**

Fifteen out of 254 women with acquired TTP were included, namely four cases with gravidic TTP, five with miscarriage, and six controls with uncomplicated pregnancy. In the cases, ADAMTS13 activity levels in the first trimester were moderately-to-severely reduced (median levels <3% in gravidic TTP and median levels 20% [range 14-40%] in the women with miscarriage) and anti-ADAMTS13 antibodies were invariably present, while in the control group ADAMTS13 activity levels were normal (median 90%, range 40-129%), with absence of detectable anti-ADAMTS13 antibodies. Reduced levels of ADAMTS13 activity (<25%) in the first trimester were associated with an over 2.9-fold increased risk for gravidic TTP and with an over 1.2-fold increased risk for miscarriage (lower boundary of the confidence interval of the odds ratio). In addition, the presence of anti-ADAMTS13 antibodies during pregnancy was associated with an over 6.6-fold increased risk for gravidic TTP and with an over 4.1-fold increased risk for miscarriage.

**Conclusions:**

ADAMTS13 activity evaluation and detection of anti-ADAMTS13 antibody could help to predict the risk of complications in pregnant women with a history of acquired TTP.

## Background

Thrombotic Thrombocytopenic Purpura (TTP) is an acute, life-threatening thrombotic microangiopathy caused by deficient activity of the von Willebrand factor (VWF) cleaving protease ADAMTS13 (*a d*isintegrin *a*nd *m*etalloprotease with *t*hrombo*s*pondin type 1 motif, 13^th^ member). The acquired form of the disease, accounting for about 95% of all cases, is caused by autoantibodies directed against the circulating metalloprotease which leads to impaired cleavage of VWF [1].

Similarly to many other autoimmune disorders, acquired TTP generally affects young women, with a peak of incidence in the third and fourth decades of life [2]. Almost half of TTP cases occur in women in childbearing age and 12-25% of acute episodes are reported in association with pregnancy or puerperium [3-7]. The association between pregnancy and TTP is supported by clinical observations and by the evidence that VWF-ADAMTS13 balance varies during normal pregnancy. VWF levels increase during the last trimester of normal pregnancy and ADAMTS13 levels progressively reduce, reaching values of as low as 25-30% in some women in the third trimester [8-10].

Since pregnancy may trigger episodes of TTP*,* pregnant women with a history of TTP are considered to be at high risk for recurrence [6,11]. In women affected by congenital TTP, a form caused by *ADAMTS13* gene mutations, almost all pregnancies are complicated by overt TTP in the absence of prophylactic plasma infusion [12,13]. For pregnant women with a history of acquired TTP, the risk of recurrence is not well established and no predictors are known. Accurate risk estimates are lacking, and no data are available on the risk of miscarriage. Jiang Y *et al.* recently reported that recurrent TTP complicating a subsequent pregnancy may be uncommon and that other complications, such as preeclampsia, may be increased [14]. These authors also reported two cases of early foetal death, on a total of 16 pregnancies. However, no estimates on risk factors were reported, and ADAMTS13 assays were only performed in a minority. Another recent study by Scully M *et al.* did also suggest that regular monitoring of ADAMTS13 activity is advisable for achieving an uncomplicated pregnancy, allowing for possible prophylactic correction [15]. However, no estimates on the predictive role of ADAMTS13 levels or anti-ADAMTS13 antibodies were reported.

With this background, the aim of this study was to evaluate factors associated with adverse gravidic outcome in women with a history of acquired TTP, in terms of TTP recurrence or miscarriage. Since TTP is a rare disease, many studies are based on selected patient series referred to highly-specialized secondary-care centres. This may result in ill-defined cohorts and estimates may be biased. To minimize referral and selection bias, we applied strict inclusion criteria and a nested case–control design.

## Methods

We conducted a nested case–control study of women who became pregnant after the diagnosis of acquired TTP. Among them, we contrasted data of women who experienced a complicated pregnancy (i.e., cases of either gravidic TTP or miscarriage) to those with an uncomplicated pregnancy (i.e., controls).

### Patients

Patients included in this study were women with a history of acquired TTP referred to Angelo Bianchi Bonomi Hemophilia and Thrombosis Centre, Fondazione IRCCS Ca’ Granda Ospedale Maggiore Policlinico, Milan (Italy), and enrolled in the Milan TTP International Registry from 1994 to October 2012, who subsequently became pregnant. In order to be included in this study, the following two criteria had to be met: (a) diagnosis of acquired TTP; (b) a first confirmed pregnancy that occurred after the diagnosis of acquired TTP and the inclusion in our Registry. TTP was defined according to internationally accepted criteria, that is by at least one episode with the following characteristics: (i) thrombocytopenia; (ii) microangiopathic haemolytic anaemia; (iii) exclusion of alternative explanations (such as the enterohaemorrhagic form of haemolytic uraemic syndrome, catastrophic anti-phospholipid antibodies syndrome, preeclampsia and related syndromes, sepsis, systemic inflammatory response syndrome, disseminated intravascular coagulation, disseminated malignancy and/or bone-marrow transplantation associated TTP-like syndrome) [16]. Congenital TTP was excluded by *ADAMTS13* gene analysis in patients with persistently low ADAMTS13 levels in remission phase, despite disappearance of detectable anti-ADAMTS13 antibodies.

Since the registry collects clinical and laboratory information on TTP patients referred from different centres and was not specifically designed for follow-up, it may be affected by loss to follow-up, which precludes the estimate of absolute risks. Therefore, the study was restricted to women for whom follow-up was available and designed as a case–control study, which allows the estimation of relative risks. We searched for any documented pregnancy, using our digital database. Specific information on pregnancies and their outcomes was obtained by medical charts or by the administration of a tailored questionnaire. The following variables were recorded: maternal age at the beginning of pregnancy, gravidity, numbers of TTP episodes before pregnancy, interval time between last TTP episode and conception, duration and outcome of pregnancy, maternal and foetal mortality, plasmatic ADAMTS13 activity levels and anti-ADAMTS13 antibodies (performed on biological samples taken during pregnancy, before any plasma infusion or exchange therapy).

Cases were women who experienced a complicated pregnancy (either gravidic TTP or miscarriage), controls those who had an uncomplicated pregnancy. Gravidic TTP was defined as an acute TTP episode occurring during pregnancy or in the post-partum period (40 days after delivery). Miscarriage was defined as naturally-occurring expulsion of the product of conception before the 20^th^ week of gestation, in the absence of thrombocytopenia and/or haemolytic anaemia.

All patients included in the Registry signed an informed consent, approved by the Ethics Committee of our Hospital, following Helsinki Declaration statements.

### Laboratory tests

*ADAMTS13 activity* was tested using a modified FRETS-VWF73 assay [17]. ADAMTS13 activity values were expressed as percentage of pooled normal plasma standard curve (arbitrarily considered as 100%). The lower limit of sensitivity was 3% of the reference standard (pooled normal plasma); the lower value of the reference interval (45%) was set at the 5^th^ percentile of the distribution of the values obtained in 72 healthy individuals.

*Anti-ADAMTS13 autoantibodies* (IgG class) were detected by Western blotting and, if positive, quantified by an ELISA assay. Western blotting was performed as reported, with minor modifications [18]. Conditioned media of cells producing recombinant ADAMTS13 (100 ng/lane) were run on 7% sodium dodecyl sulphate polyacrylamide gel electrophoresis and then were transferred onto pure nitrocellulose membrane (GE Healthcare, Buckinghamshire, UK). Thereafter, membranes were incubated with citrated plasma (diluted 1/100) of TTP patients as source of anti-ADAMTS13 antibody. Bound antibodies were visualized using horseradish peroxidase-labelled anti-human IgG (Sigma, St. Louis, MO, USA) and the Opti-4CN substrate kit (BioRad, Hercules, CA, USA). With regards to the ELISA assay [19], a reference curve was obtained by serially diluting the plasma of a TTP patient with a high anti-ADAMTS13 IgG titre; the level of anti-ADAMTS13 IgG in sample plasmas was then calculated as a percentage of the reference curve (arbitrarily assigned a value of 100%). The cut-off for the normal reference range was calculated on 74 healthy subjects, and values higher than 2 standard deviations were considered positive (anti-ADAMTS13 IgG > 1.18%).

### Statistical analysis

Descriptive statistics were used for demographic and clinical characteristics in each group.

We used odds ratios (OR), as an approximation of relative risks for the variables of interest. The 95% confidence interval (CI) of the OR was calculated according to Woolf based on Poisson distributions and, when appropriate, to Cornfield’s approximate method [20].

The following variables were studied: age at pregnancy (less than 30 years *versus* older); gravidity (primigravidae *versus* multigravidae); TTP recurrence (non-recurrent TTP *versus* recurrent); time from the last TTP episode (conception within 24 months from the previous episode of TTP *versus* more than 24 months); ADAMTS13 activity levels (less than 25% *versus* higher); anti-ADAMTS13 antibody (presence *versus* absence).

With regards to ADAMTS13-related measurements, we contrasted cases and controls for ADAMTS13 activity levels in the first trimester (before any plasma infusion or exchange therapy), for the following reasons: (i) in the miscarriage group, pregnancy ended in the first trimester; (ii) in the gravidic TTP cases, once severe ADAMTS13 deficiency was detected, prophylactic treatment was started thus subsequent samples were unsuitable for analysis. The cut-off of 25% for ADAMTS13 activity levels was chosen because below this value patients need closer monitoring since they are approaching the critical level of 10% which is associated with the risk of TTP recurrence.

## Results

**Figure 1:**
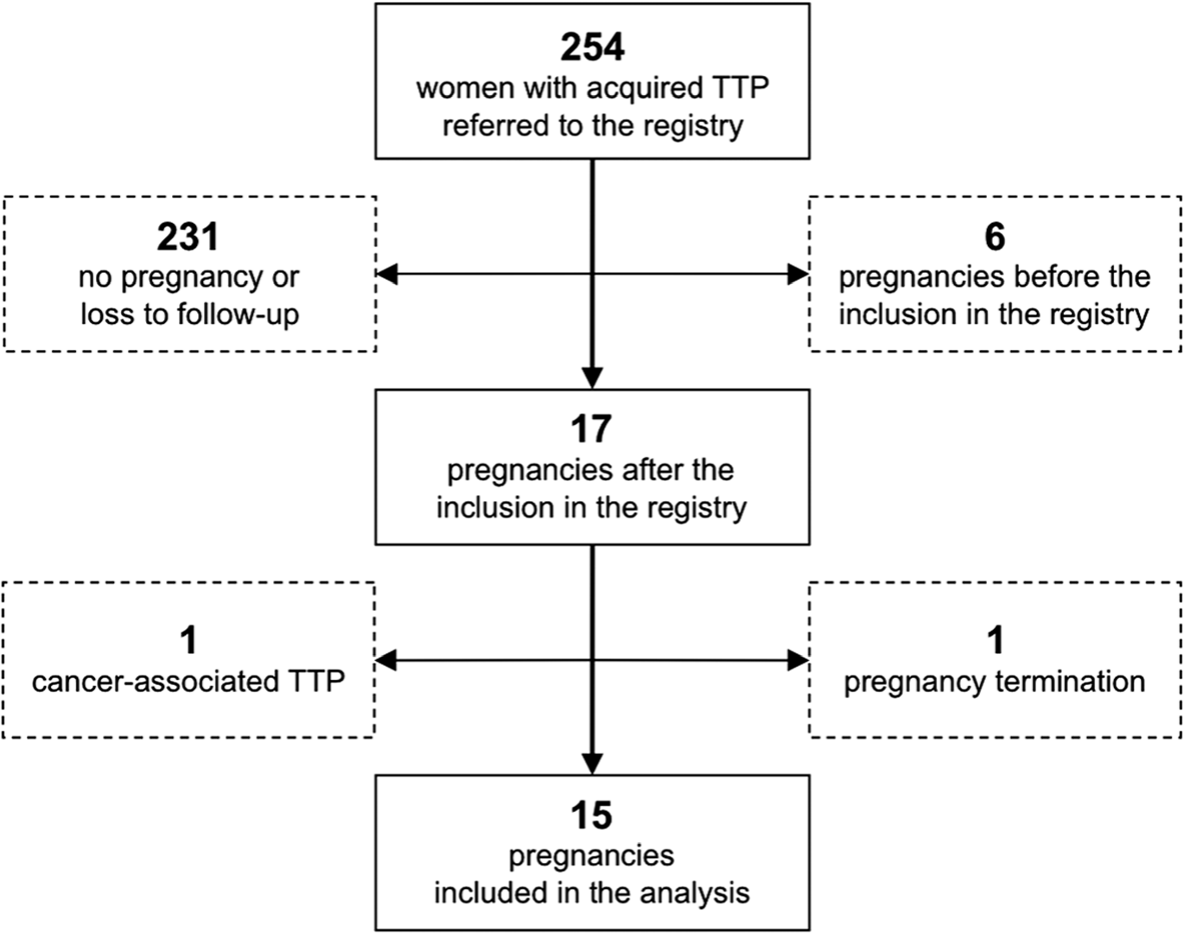
**Study flow-chart for women selection.** *Abbreviations: TTP*, Thrombotic thrombocytopenic purpura.

At the moment of the closing of the database (October 2012), the Milan TTP Registry included 254 women with acquired TTP; of those, 17 met the strict inclusion criteria of this study. One patient was excluded because of pregnancy termination (reason unrelated to TTP) and another one was excluded due to a history of cancer-associated recurrent TTP. Therefore 15 women were included in the analysis (Figure 1). Their main clinical characteristics (age at pregnancy, gravidity, pregnancy outcome and duration, number of previous episodes of TTP) are reported in Table 1, while ADAMTS13-related measurements are reported in Table 2. All women were Caucasian; median age at pregnancy was 32 years (range 24–38 years) and 9 were primigravidae; 6 had a history of recurrent TTP; the median time between the previous TTP episode and conception was 28 months (range 2–107 months). All patients were affected by autoimmune TTP (detectable anti-ADAMTS13 antibodies at least once in the patient’s history, before inclusion in the study); in all women except patient 11, severe ADAMTS13 deficiency (i.e., <10%) was detected in either acute or remission phase, out of pregnancy.

**Table 1:** **Demographic and clinical characteristics of women included in the study**

**Patient**	**Maternal age (years)**	**Gravidity**	**Outcome of pregnancy (onset, weeks)**	**Duration of pregnancy (weeks)**	**Previous TTP episode (gravidic)**
1	26	M	Gravidic TTP (21)	30	1 (1)
2	30	P	Gravidic TTP (*na*)	*na*	1
3	34	M	Gravidic TTP (34)	34	2 (1)
4	24	P	Gravidic TTP (30)	31	1
5	36	M	Miscarriage	10	2 (1)
6	38	M	Miscarriage	9	1 (1)
7	26	M	Miscarriage	6	1
8	32	P	Miscarriage	3	1
9	32	P	Miscarriage	8	1
10	36	P	Uncomplicated	38	3
11	32	P	Uncomplicated	37	3
12	29	P	Uncomplicated	39	1
13	29	M	Uncomplicated	38	1
14	34	P	Uncomplicated	38	2
15	33	P	Uncomplicated	36	4

*Abbreviations:* *M* Multigravida, *P* Primigravida, *TTP* Thrombotic thrombocytopenic purpura, *na* Not available.

**Table 2:** **ADAMTS13 activity levels and anti-ADAMTS13 antibodies during pregnancy of women included in the study**

**Patient**	**ADAMTS13 activity, by FRET**			**Anti-ADAMTS13 IgG, by WB (%, by ELISA)**		
	**1 T**	**2 T**	**3 T**	**1 T**	**2 T**	**3 T**
1	<3	<3	*on PEX*	P (4)	P (5)	*on PEX*
2	<3	*on PEX*		P (3)	*on PEX*	
3	na	na	<3	na	na	P (7.5)
4	na	na	6	na	na	P (3.5)
5	40	*Miscarriage*		P (2)	*Miscarriage*	
6	na	*Miscarriage*		na	*Miscarriage*	
7	14	*Miscarriage*		P (4.4)	*Miscarriage*	
8	na	*Miscarriage*		na	*Miscarriage*	
9	20	*Miscarriage*		P (<1.18)	*Miscarriage*	
10	129	97	70	N	N	N
11	40	39	45	N	N	N
12	90	84	80	N	N	N
13	na	na	73	na	na	N
14	64	96	68	N	N	N
15	90	na	na	N	na	na

*Abbreviations:* *WB* Western blotting, *T* Trimester, *P* Positive, *N* Negative, *PEX* Plasma-exchange, *na* Not available.

According to pregnancy outcome, the cases were divided in two groups, cases of gravidic TTP (n = 4) and cases of miscarriage (n = 5), while women with uncomplicated pregnancy served as controls (n = 6). The main clinical characteristics of cases and controls are reported in Table 3.

**Table 3:** **Demographic and clinical characteristics of women according to pregnancy outcome**

	**Gravidic TTP (n = 4)**	**Miscarriage (n = 5)**	**Uncomplicated pregnancy (n = 6)**
Maternal age at pregnancy, median [range], years	28 [24–34]	32 [26–38]	33 [29–36]
Primigravidae	2	2	5
History of recurrent TTP (>1 episode)	1	1	4
Months since last TTP episode, median [range]	29 [20–107]	17 [2–38]	35 [5–70]
ADAMTS13 activity (%) in the first trimester, median [range] (available)	<3 (2)	20 [14–40] (3)	90 [40–129] (5)
Positive anti-ADAMTS13 antibodies in the first trimester (available)	2 (2)	3 (3)	0 (5)
Positive anti-ADAMTS13 antibodies in any trimester (available)	4 (4)	3 (3)	0 (6)

**Figure 2:**
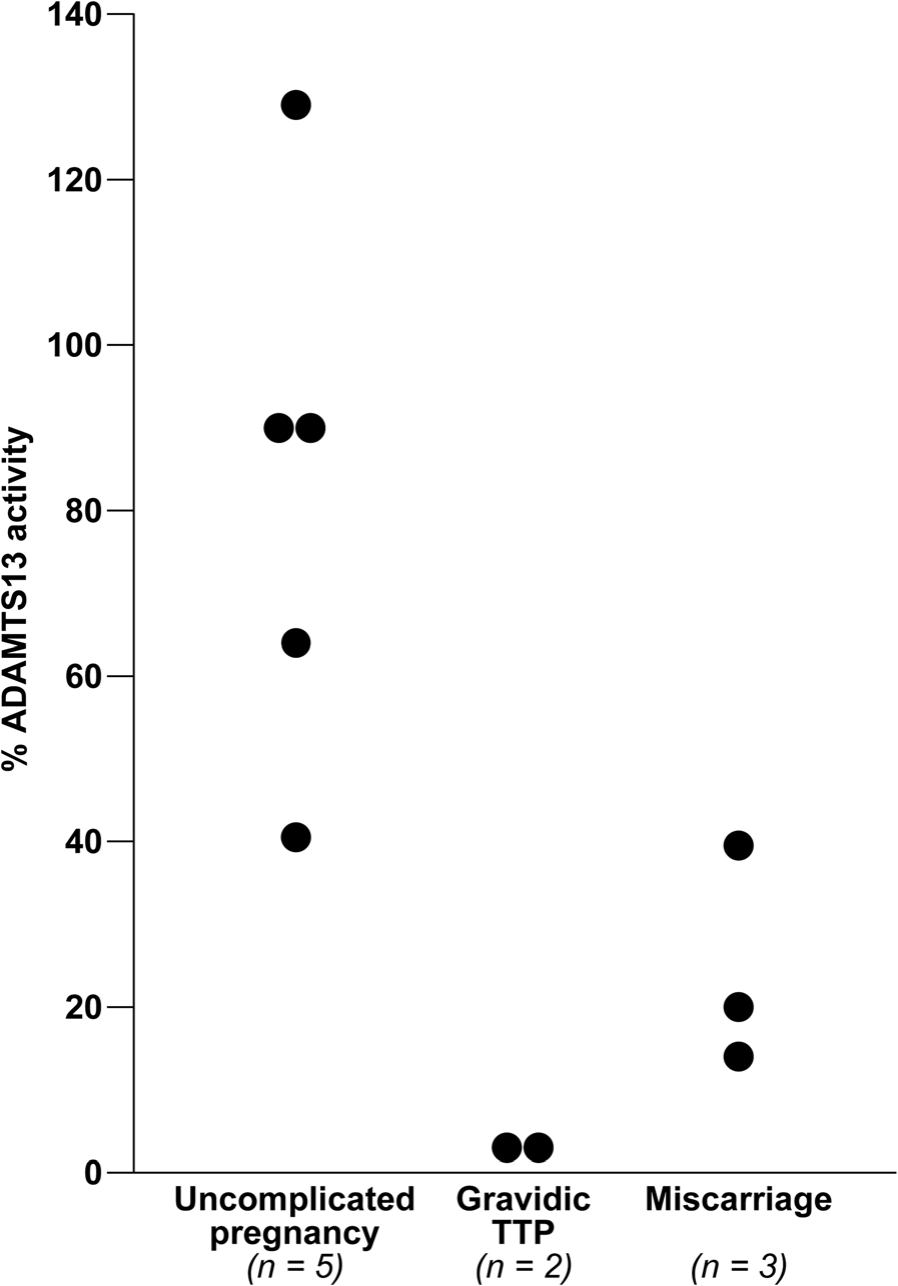
**ADAMTS13 activity levels in the first trimester according to pregnancy outcomes.** *Abbreviations: TTP*, Thrombotic thrombocytopenic purpura. For women with multiple tests, mean values are reported.

Gravidic TTP recurrences developed in the second or third trimester, while all five miscarriages occurred in the first trimester (i.e., by 13^th^ +1 gestational week). ADAMTS13 activity levels in the first trimester were significantly reduced in the cases of gravidic TTP, with median levels <3%, while ADAMTS13 deficiency was less severe in the cases of miscarriage, with median levels of 20% (range 14-40%). Anti-ADAMTS13 antibodies resulted to be invariably present in either group of cases, with median anti-ADAMTS13 IgG levels of 4% (range 3–7.5%) in women with gravidic TTP and 2% (range **<**1.18-4.4%) in the miscarriage group. In the control group of women with uncomplicated pregnancies ADAMTS13 activity levels were normal in the first trimester (median 90%, range 40-129%) and remained above 39% until delivery, with absence of detectable anti-ADAMTS13 antibodies (Figure 2).

Tables 4 and 5 show the odds ratios for the variables that were included, indicating the associated relative risk of a complicated pregnancy (either gravidic TTP or miscarriage). The highest odds ratios were associated with reduced ADAMTS13 activity levels (below 25%) in the first trimester, with lower boundary of the confidence interval of the odds ratio over 2.9 for gravidic TTP (OR ∞, 95% CI 2.9 to ∞) and over 1.2 for miscarriage (OR ∞, 95% CI 1.2 to ∞), and with the presence of anti-ADAMTS13 antibodies during pregnancy, with lower boundary of the confidence interval of the odds ratio over 6.6 for gravidic TTP (OR ∞, 95% CI 6.6 to ∞) and over 4.1 for miscarriage (OR ∞, 95% CI 4.1 to ∞). We found that young women (<30 years) had a higher risk for gravidic TTP than older women (OR 6.0, 95% CI 0.4 to 101.6). Primigravidae had a lower risk for both gravidic TTP (OR 0.2, 95% CI 0.01 to 3.7) and miscarriage (OR 0.1, 95% CI 0.01 to 2.2) than multigravidae. Women with a single previous episode of TTP were at higher risk for both gravidic complications than women with multiple TTP events (gravidic TTP OR 6.0, 95% CI 0.4 to 101.6; miscarriage OR 8, 95% CI 0.5 to 127.9). Conception before 24 months from the previous TTP episode conferred a highly elevated risk for miscarriage (OR 20, 95% CI 0.9 to 429.9).

**Table 4:** **Risk estimates for gravidic TTP associated with demographic and clinical variables**

	**Gravidic TTP (n = 4)**	**Uncomplicated pregnancy (n = 6)**	**OR (95% CI)**
Age ≤ 30 years	3	2	6.0 (0.4 – 101.6)
Age >30 years	1	4	ref
Primigravidae	2	5	0.2 (0.01 – 3.7)
Multigravidae	2	1	ref
Non-recurrent TTP	3	2	6.0 (0.4 – 101.6)
Recurrent TTP	1	4	ref
Time from previous TTP ≤24 months	1	1	1.7 (0.08 – 37.7)
Time from previous TTP >24 months	3	5	ref
ADAMTS13 activity <25% in the first trimester*	2	0	∞ (2.9 - ∞)
ADAMTS13 activity ≥25% in the first trimester*	0	5	ref
Positive anti-ADAMTS13 antibodies in any trimester	4	0	∞ (6.6 - ∞)
Negative anti-ADAMTS13 antibodies in any trimester	0	6	ref

*ADAMTS13 activity levels in the first trimester were available in 2 cases and 5 controls.

**Table 5:** **Risk estimates for miscarriage associated with demographic and clinical variables**

	**Miscarriage (n = 5)**	**Uncomplicated pregnancy (n = 6)**	**OR (95% CI)**
Age ≤ 30 years	1	2	0.5 (0.03 – 8.06)
Age >30 years	4	4	ref
Primigravidae	2	5	0.1 (0.01 – 2.2)
Multigravidae	3	1	ref
Non-recurrent TTP	4	2	8.0 (0.5 – 127.9)
Recurrent TTP	1	4	ref
Time from previous TTP ≤24 months	4	1	20.0 (0.9 – 429.9)
Time from previous TTP >24 months	1	5	ref
ADAMTS13 activity <25% in the first trimester*	2	0	∞ (1.2 - ∞)
ADAMTS13 activity ≥25% in the first trimester*	1	5	ref
Positive anti-ADAMTS13 antibodies in the first trimester*	3	0	∞ (4.1 - ∞)
Negative anti-ADAMTS13 antibodies in the first trimester*	0	5	ref

*ADAMTS13 activity levels and anti-ADAMTS13 antibodies in the first trimester were available in 3 cases and 5 controls.

None of the 15 women received regular prophylactic plasma infusion or exchange during gestation, as part of a planned pregnancy; prophylactic plasma-exchange was performed after detection of severely deficient ADAMTS13 activity levels (i.e., <10%) in patients 1 and 2 but, nevertheless, they developed gravidic TTP. Almost half of the cases (2/4 cases of gravidic TTP and 2/5 cases of miscarriage) have had a history of previous gravidic TTP, in contrast with none of the controls.

None of the 15 women had a history of poliabortivity and, among the cases of miscarriage, only one woman had had a single previous miscarriage (before the first TTP episode).

The outcome of pregnancy in the complicated pregnancies was poor, for in one case both the mother and her foetus died during TTP recurrence (patient 2); six foetuses died, due to miscarriage occurring either before or during gravidic TTP (patient 2).

## Discussion

We conducted a study on clinical factors associated with adverse gravidic outcome in women with a history of acquired TTP. To avoid bias associated with studies on rare disorders conducted in referral centres and registries, we applied the strictest inclusion criteria and a nested case–control design.

The strongest predictors of poor gravidic outcome were reduced levels of ADAMTS13 activity below 25% in the first trimester and the presence of anti-ADAMTS13 antibodies, both for gravidic TTP recurrence and miscarriage. Published data from case series and one prospective cohort study have suggested that low levels of ADAMTS13 are associated with poor pregnancy outcome, but no risk estimates were available [15,21-25]. Moreover, anti-ADAMTS13 antibodies are the hallmark of autoimmune TTP but their predictive role in remission remains unclear, particularly in relation to added triggers, such as pregnancy [18,26].

In our study, all the cases showed a moderate to severe reduction of ADAMTS13 activity levels in association with anti-ADAMTS13 antibodies before the gravidic complication occurred. On the contrary, women with uncomplicated pregnancies showed near-normal ADAMTS13 activity levels till delivery, with no anti-ADAMTS13 antibodies. Our results suggest that low ADAMTS13 activity levels (<25%) in the first trimester in the presence of anti-ADAMTS13 antibodies represent not only a risk factor for TTP recurrence, but also for miscarriage. It is well known that early changes in the maternal immunity system are crucial for pregnancy outcome, since immune-tolerance toward the semi-allogeneic foetus is necessary for physiologic progression of any gestation [27]. If this process does not work correctly, gestation might be interrupted. Women affected by other systemic autoimmune diseases (such as systemic lupus erythematosus and systemic sclerosis) have an increased risk of miscarriage and such risk is associated with the activity of the disease. Whether immune system dysregulation in acquired autoimmune TTP and the presence of anti-ADAMTS13 antibodies might be responsible for increased risk of miscarriage in these women, needs to be further investigated. In our study miscarriage and gravidic TTP were exclusive outcomes, which may be the result of women with early miscarriage no longer being at risk to develop gravidic TTP.

The results of this study support the hypothesis that ADAMTS13 activity levels and anti-ADAMTS13 antibodies are useful prognostic markers for gravidic complications (TTP recurrence and miscarriage) in women with previous acquired TTP. Therefore, we suggest to strictly monitor all pregnant women with a history of acquired TTP for both markers before and regularly during their pregnancy. For all other variables included in this study (age at pregnancy, gravidity, TTP recurrence, time from the last TTP episode), the preliminary results need to be further investigated.

Our study has some limitations. The number of pregnancies was limited, due to the strict design of the study, and the low sample size renders many estimates imprecise. However, our choice of study design resulted in enhanced rigour and these estimates, despite less precise, are also less biased. Moreover, we note that this was one of the largest studies to date on the issue of TTP-related gravidic complications and we are the first to present risk estimates for this rare devastating disorder. Since we are a tertiary-care centre for acute TTP, uncomplicated pregnancies and miscarriages may not have been referred. Our nested case–control design was specifically aimed at reducing ensuing referral and selection bias. Data on ADAMTS13 activity levels and anti-ADAMTS13 antibodies during pregnancy were not available for all women.

## Conclusions

Reduced ADAMTS13 activity levels and the presence of anti-ADAMTS13 antibodies represent risk factors for poor outcome of pregnancy in women with a history of acquired TTP. Given the desire of maternity in many women affected by TTP at a young age, and the potentially severe consequences of TTP during pregnancy, these results may be useful to tailor future therapeutic strategies in these women.

## Availability of supporting data

The data set supporting the results of this article is not available in a publicly-accessible data repository but will be made available after request to the corresponding author.

## Abbreviations

TTP: Thrombotic thrombocytopenic purpura
VWF: Von Willebrand factor
OR: Odds ratio
CI: Confidence interval
